# Structural and dielectric properties of electrospun P(VDF-TrFE) and P3HT filled with copper oxide nanofillers

**DOI:** 10.1038/s41598-025-90461-x

**Published:** 2025-03-04

**Authors:** E. Salim, M. I. El-Henawey

**Affiliations:** ESCs Research Lab, Physics Department, Faculty of Science, Mansoura University, Mansoura, Egypt

**Keywords:** P(VDF-TrFE), P3HT, CuO, Electrospinning, Dielectric constant, Materials science, Nanoscience and technology, Physics

## Abstract

Nanofibers based on polyvinylidene fluoride-trifluoroethylene/poly(3-hexylthiophene) (P(VDF-TrFE)/P3HT) containing copper oxide nanoparticles (CuO NPs) were fabricated using the electrospinning technique. Scanning electron microscope (SEM) images revealed smooth, bead-free solid nanofibers of 34–100 nm diameters. X-ray diffraction (XRD) analysis shows the ferroelectric β phase of P(VDF-TrFE) with a wide diffraction pattern. Fourier transform infrared (FTIR) spectrum of the P(VDF-TrFE) fiber film revealed distinct peaks for the polar β phase, aromatic C–H groups, and alkyl groups. Semiconducting P3HT/CuO increases the dielectric constant of P(VDF-TrFE), inducing polarization at the semiconductor-insulator interface. Two types of dielectric response are observed: β-phase rotation and α to β phase transition. The bulk resistance values show a reduction from 224.5 to 23.8 Ω, suggesting more conductive pathways. Furthermore, incorporating P3HT/CuO into P(VDF-TrFE) enhanced its electrical conductivity from 3.75 × 10^3^ to 3.40 × 10^4^ S m^−1^. This investigation showed that P3HT/CuO-enhanced P(VDF–TrFE) is a viable option for energy harvesting.

## Introduction

Over the past decade, there has been significant research focus on polymers and polymer nanocomposites due to their wide-ranging applications in the domains of electronics and solar cells^1–4^. Polyvinylidene fluoride (PVDF) and its copolymer polyvinylidene fluoride-trifluoroethylene (PVDF-TrFE) are commonly employed in electronic applications due to their excellent properties. PVDF has a semicrystalline structure, composed of a repeating –CH_2_CF_2_ monomer^5,6^. The high electronegativity of the fluorine and hydrogen atoms in PVDF causes the dipole moment. The orientation of dipoles in PVDF is precisely determined by the molecular conformation and packaging of its constituent molecules during crystallization^7,8^. PVDF monomers may produce trans (T) or gauche (G) linkages depending on crystallization conditions. The crystalline structures of PVDF have been shown in several reports, with at least five crystal phases: α, β, γ, ε, and δ^9^. β-phase, with its all-trans planar zigzag conformation and fluorine atoms on the same side of the polymer chains, provides PVDF a larger net dipole moment than other PVDF phases^10^. Incorporating TrFE, a three-fluoride monomer, into PVDF copolymerization may improve the all-trans conformation of the *β* phase. Fluorine atoms from TrFE monomer can hinder molecular structuring due to their bulkiness. Additionally, the copolymer containing TrFE exhibits higher crystallinity than pure PVDF when annealed. P(VDF-TrFE) with a molar ratio of 70/30 exhibits a greater spontaneous polarization of about 10 µC/cm^2,11,12^.

As the conducting material for this investigation, the organic semiconductor P3HT, whose prospective applications in solar cells^13,14^ and transistors^15^ have generated significant interest, was chosen. The selection of P3HT was based on its exceptional environmental and thermal stability, high electrical conductivity, and ability to be processed in solution. Moreover, adding inorganic compounds to polymeric materials is a common method for improving their physical, functional, and mechanical properties. Common metal oxides used to enhance the structural and dielectric characteristics of P(VDF-TrFE) like CuO^16^ and zinc oxide (ZnO)^17^. Here, CuO is selected as a filler due to its versatility in a variety of applications^18^. CuO NPs can be used in photodetectors, magnetic storage, photocatalysis, supercapacitors, and solar cells^19,20^. PVDF-TrFE, P3HT, and CuO are compatible with electrospinning due to their simplicity of processing, excellent charge mobility, electroactivity, and multifunctionality, respectively. Suresh et al.^16^ measured the dielectric permittivity of P(VDF-TrFE)-CuO nanocomposite solutions in the microwave frequency range using an open-ended coaxial probe technique. Results showed that increasing CuO concentration decreases dielectric permittivity while increasing CuO concentration increases dielectric constant. The study also analyzed the dielectric relaxation time and calculated optical, static, and molecular relaxation times. The object of this study is to fabricate polymeric nanofibers (P(VDF-TrFE)/P3HT) loaded with CuO nanoparticles to be used in a variety of applications. The structural characteristics of the fabricated nanofiber films were examined using XRD, FTIR, UV-Vis, and scanning electron microscope (SEM). A further investigation has been conducted into the electrical, and dielectric properties of the electrospun films.

## Experimental section

### Materials

The P(VDF-TrFE) copolymer, consisting of 75 mol% VDF and 25 mol% TrFE, and with a molecular weight (Mw) of 350 mol kg^−1^, was obtained from Solvay, India. The regioregular poly(3-hexylthiophene-2,5-diyl) (P3HT), CuO nanopowder (with a particle size less than 50 nm and a trace metals base of 99.8%), and tetrahydrofuran (THF) were acquired from Sigma–Aldrich.

### P(VDF-TrFE)/P3HT/CuO fabrication

Using the electrospinning technique, P(VDF-TrFE) nanofibers and composites containing P3HT and P3HT/CuO were fabricated. The P(VDF-TrFE) nanofibers were initially electrospun with a 13 wt% concentration of P(VDF-TrFE) in a THF solution. Second, to produce the composite solutions, two mixtures of 2 wt% P3HT and P(VDF-TrFE) in a THF solution were continuously stirred. Furthermore, one of the P(VDF-TrFE)/P3HT solutions was combined with 3 mg of CuO dispersed in 1 ml THF as the third solution. All nanofibers were formed onto aluminum foil by electrospinning the composite solutions at 12 kV, 0.2 ml/h pump speed, and a distance of 13 cm between the needle and collector. Electrospinning was performed at a temperature of 21 °C and a relative humidity of 25%. A humidifier was used to supply humidity within the electrospinning box.

### Characterization

The DIANO X-Ray Diffractometer was used to analyze XRD patterns using CuKα radiation. Nanofiber films’ optical absorption was measured using a UV-visible spectrophotometer (JASCO V-630-Japan). Nanofiber infrared spectra were measured using Nicolet iS10 FTIR in the 400–4000 cm^− 1^ wavenumber. The QUANTA FEG 250 SEM was used to analyze the nanofibers’ surface and microstructure. CuO nanoparticle size was examined using a TEM (JEOL/JEM/1011, Japan). The nanofiber films’ dielectric characteristics and impedance were studied at 300 K using broadband dielectric spectroscopy (Novo Control Turnkey Concept 40 System).

## Results and discussion

### SEM and TEM analysis

**Fig. 1:**
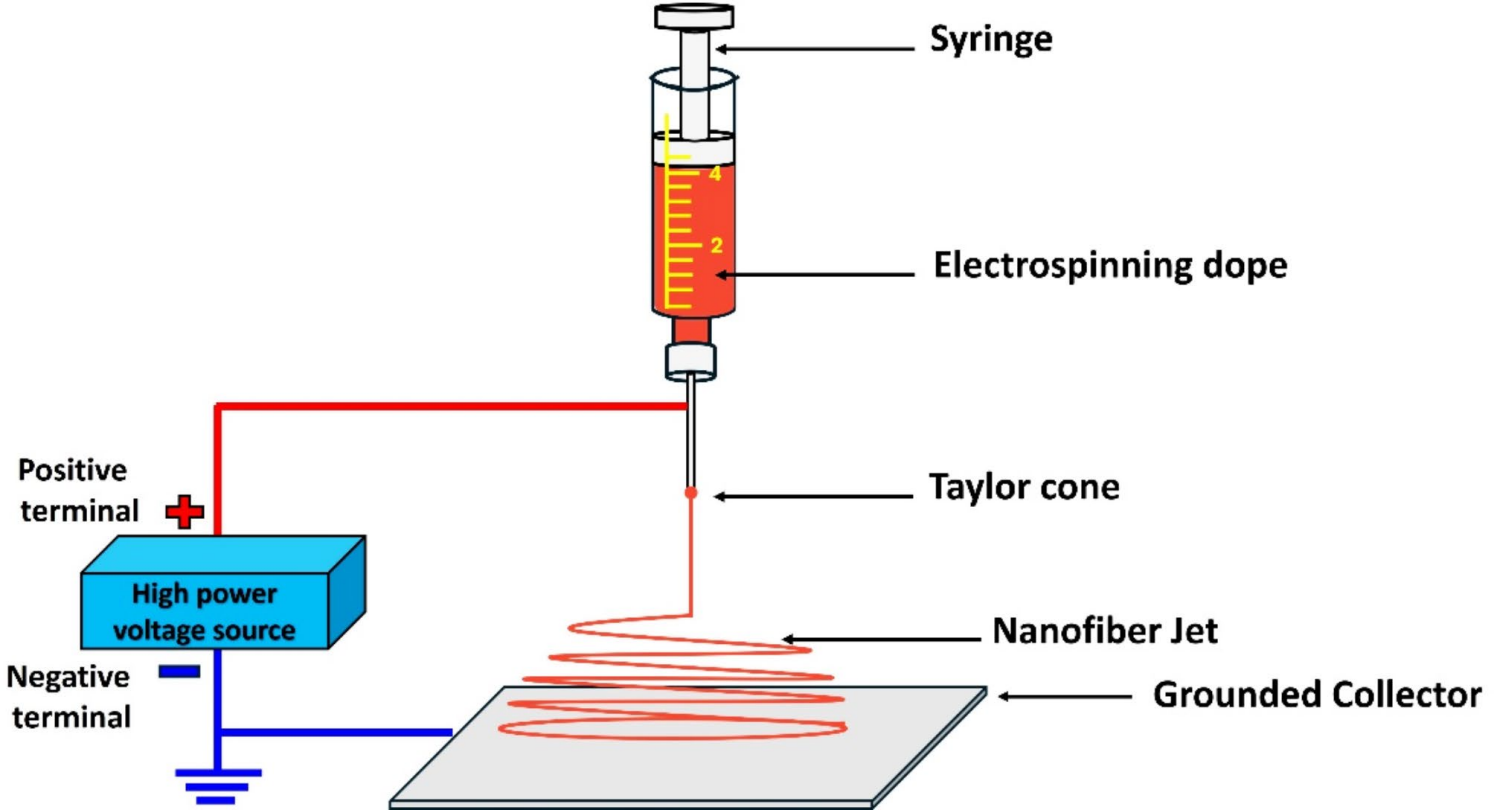
Electrospinning setup for nanofiber production.

**Fig. 2:**
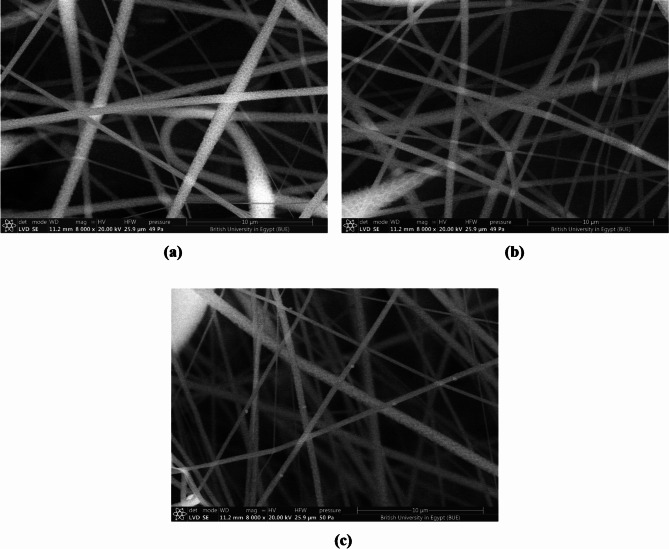
SEM images of electrospun nanofibers (**a**) P(VDF-TrFE), (**b**) P(VDF-TrFE)/P3HT, and (**c**) P(VDF-TrFE)/P3HT/CuO.

**Fig. 3:**
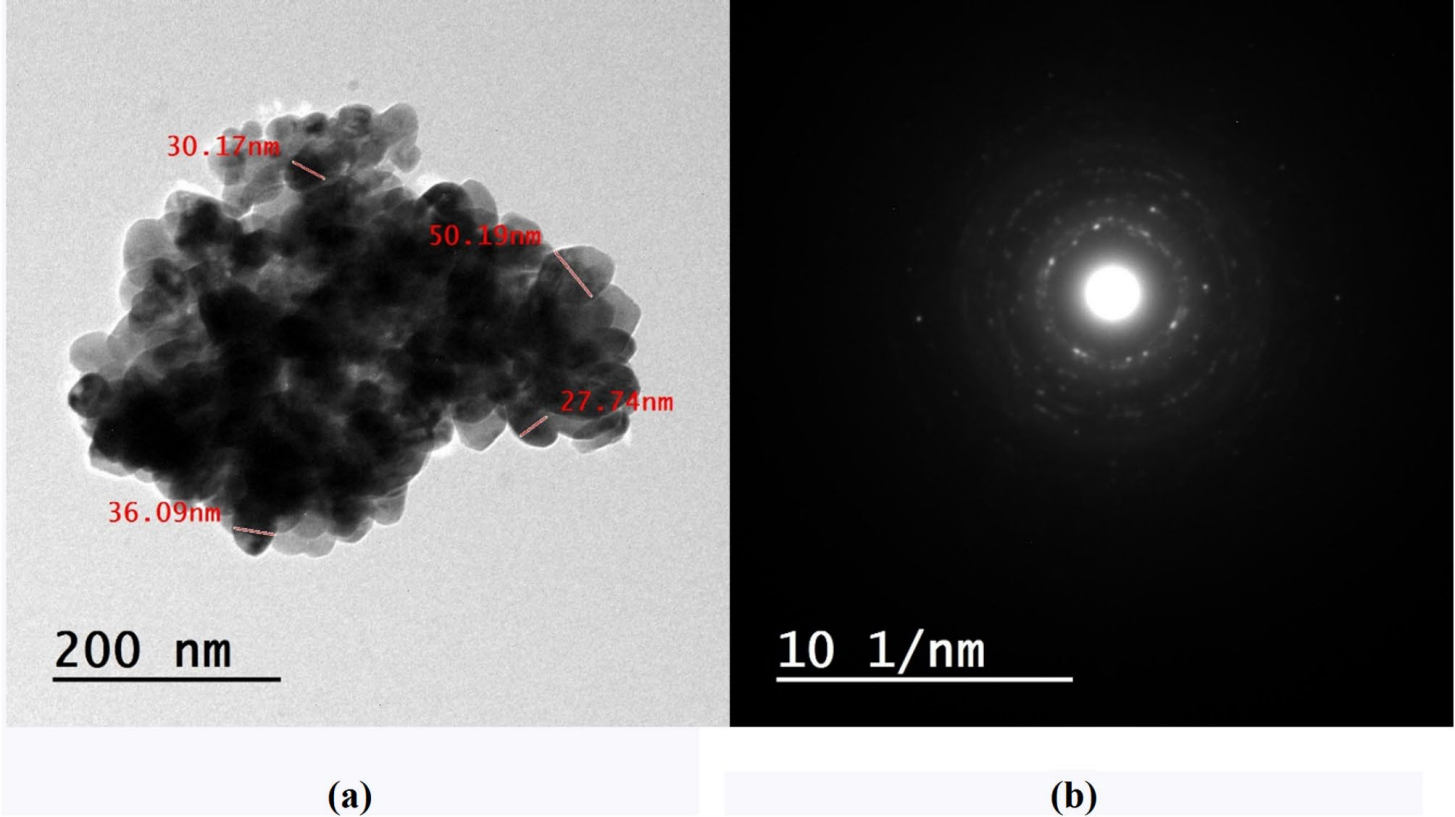
(**a**) TEM image and (**b**) SAED of CuO nanoparticles.

The semiconductive nanofiber coatings, with a thickness of 10 μm, were generated using conventional electrospinning techniques, as seen in Fig. 1. The SEM presented in Fig. 2a, b, and c illustrate P(VDF-TrFE), P(VDF-TrFE)/P3HT, and P(VDF-TrFE)/ P3HT/ CuO nanofibers that were electrospun onto aluminum foil. Smooth and bead-free solid nanofibers were formed by randomly assembling the fibers in a network. The nanofibers typically had a diameter ranging from 34 nm to 100 nm. Figure 3a show CuO NPs’ TEM image. This image shows almost sphere-like, and randomly dispersed CuO NPs with crystal sizes between 27.74 and 50.19 nm. The Selected Area Electron Diffraction (SAED) pattern in Fig. 3b further indicates the high degree of crystallinity of CuO NPs.

### X-ray analysis

**Fig. 4:**
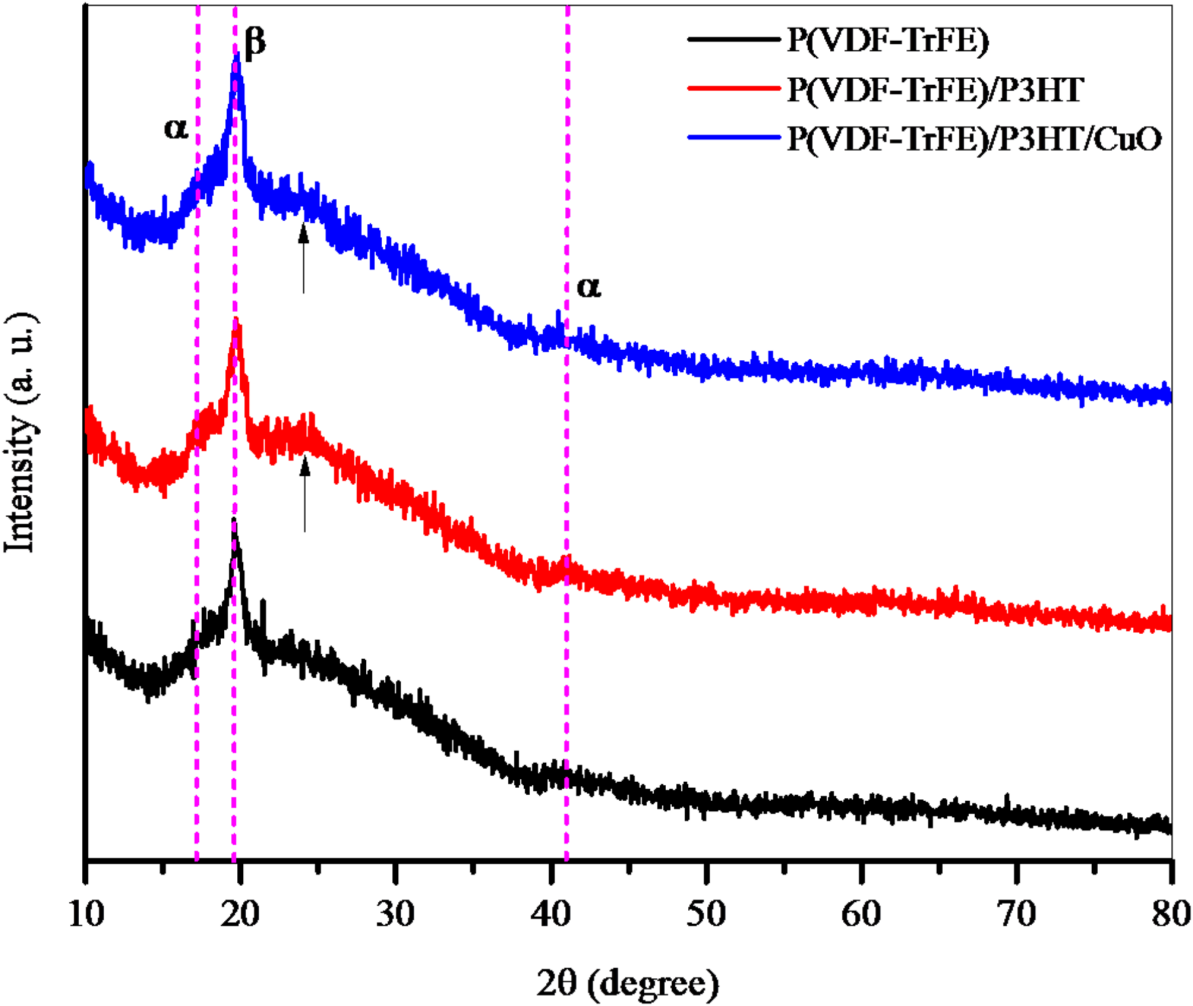
XRD patterns of electrospun nanofibers.

The crystal structure and long-range order of both random and aligned networks can be inferred from XRD patterns. The pattern of grazing incidence XRD that is displayed in Fig. 4 demonstrates the presence of the ferroelectric β phase of P(VDF-TrFE). It is consistent with the (100) and (200) planes that the peak that is centered at 2$\:{\uptheta\:}$ = 19.7° yields an interplanar distance of 4.5 Å, which is in line with previous results^21,22^. P3HT exhibits a broad diffraction pattern within the angle range of 2$\:{\uptheta\:}$ = 23° to 28°, which may be indexed as (010)^23^.

### FTIR spectroscopy

**Fig. 5:**
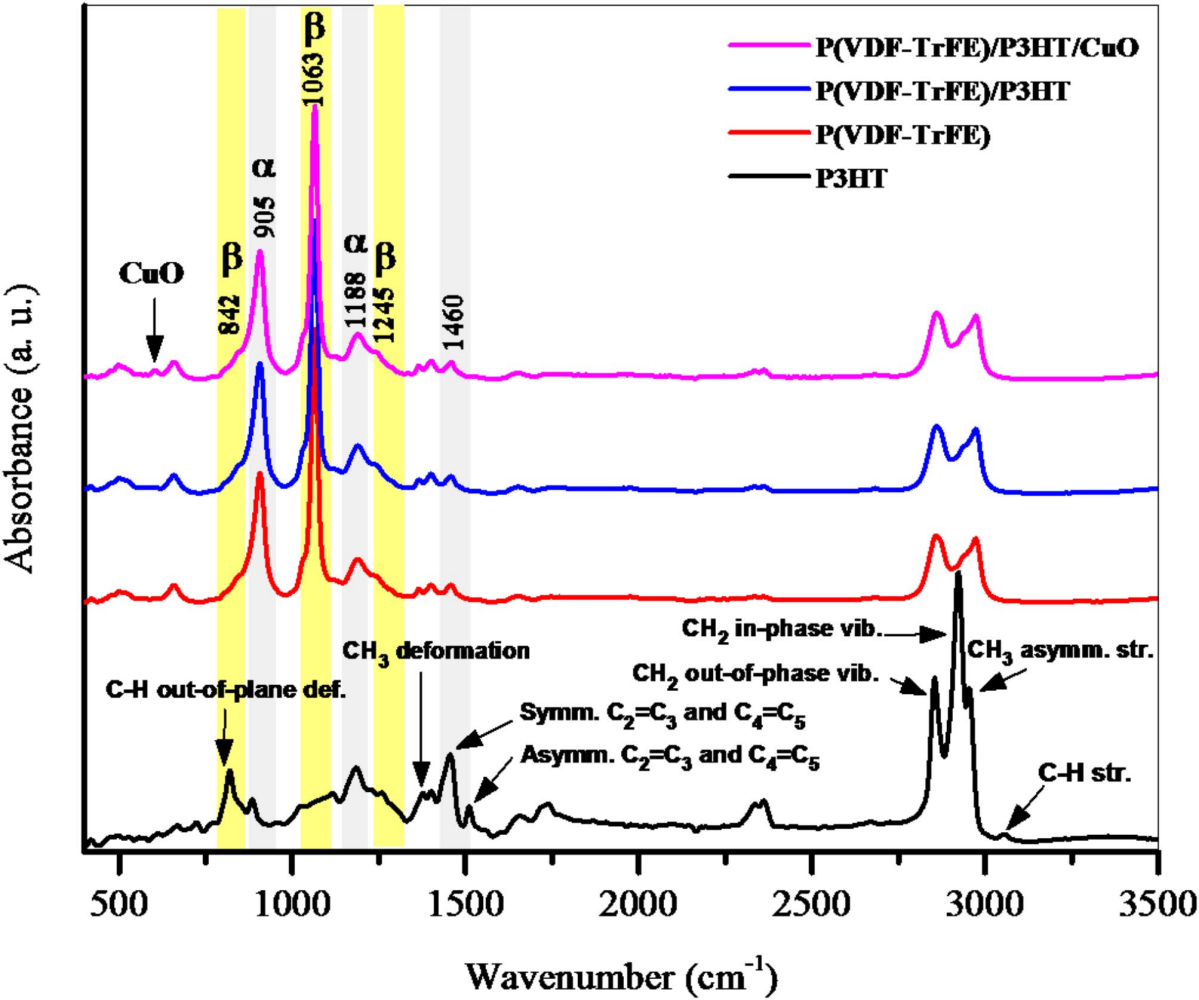
Fourier transform infrared (FTIR) spectra of electrospun of P(VDF-TrFE), P3HT, P(VDF-TrFE)/ P3HT, and P(VDF-TrFE)/P3HT/CuO nanofibers.

Figure 5 shows the FTIR spectrum of the pristine P(VDF-TrFE) fiber film exhibited distinct peaks that corresponded to its polar β phase at 842, 1063, and 1245 cm^− 1^. Additionally, the non-polar α phase was seen at 905 and 1188 cm^− 1^^24^. The main spectrum characteristics of our pristine P3HT include peaks at 1513 and 1451 cm^− 1^, which are characteristic of the thiophene ring; 3053 and 822 cm^− 1^, which are attributed to aromatic C–H groups; and 2950 to 2850 cm^− 1^, which are indicative of alkyl groups^25^. There were indications for C=C symmetric stretching at 1460 cm^− 1^ and C–C intra-ring stretching at 1375 cm^− 1^ that dominated the P(VDF-TrFE)/P3HT nanofiber film. These bands were compatible with π-electron delocalization^26^. There is also a new diffraction peak that appears at 603 cm^− 1^, which might be linked to the vibrations of the Cu-O bond^27^.

### UV–Vis absorbance

**Fig. 6:**
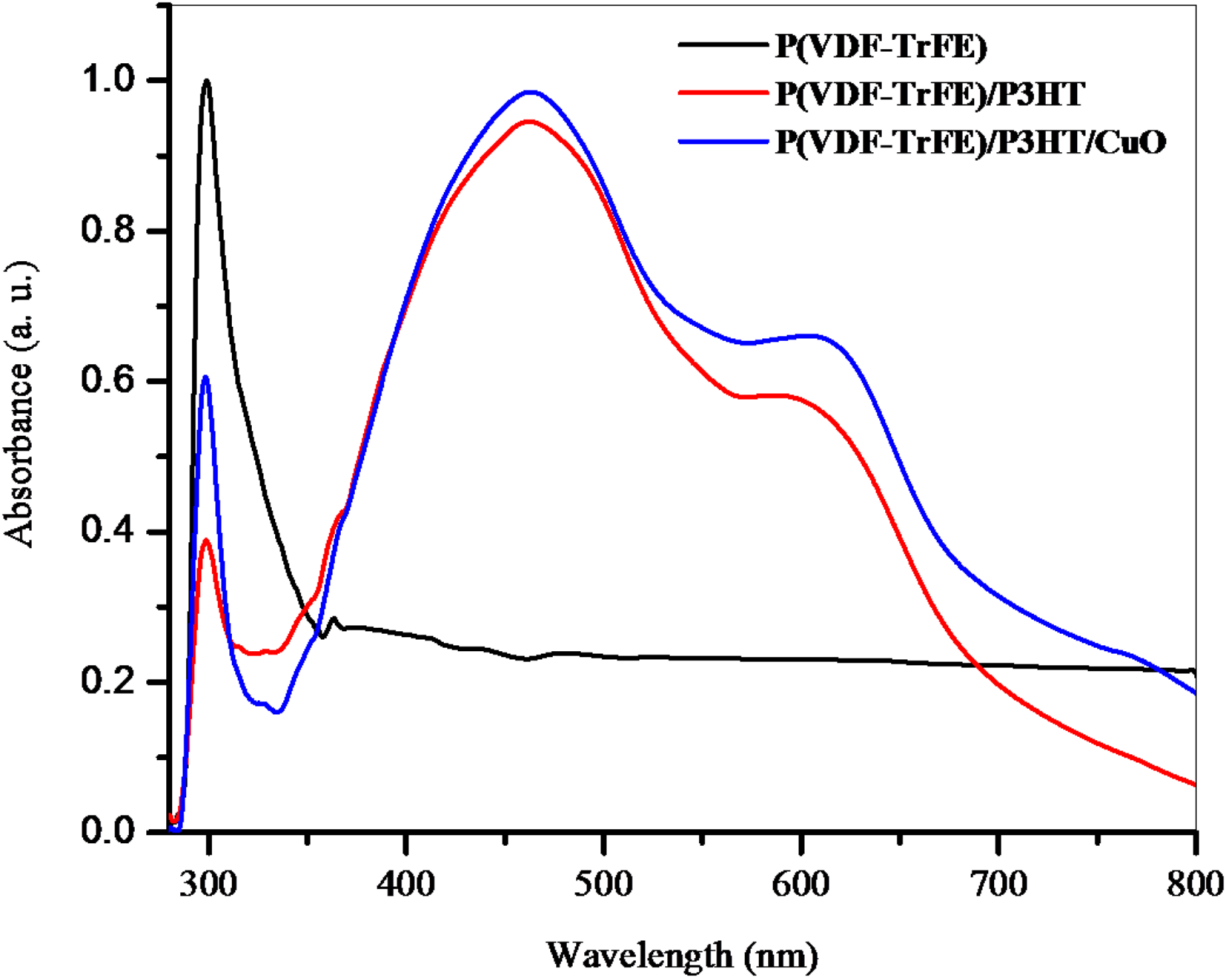
UV-visible spectra of electrospun nanofibers.

Figure 6 shows the UV-Vis spectra of the nanofibers that were electrospun in the 190–800 nm wavelength range. The absorption peak observed at a wavelength of 298 nm indicated the presence of semicrystalline P(VDF-TrFE)^28^. The spectrum of the P(VDF-TrFE)/P3HT has a wide absorption peak at around 440 nm, which is a consequence of the transition from π to π* in the electronic absorption spectra^29^. A small absorption edge is also noticed at around 600 nm is due to P3HT π-stacking, indicating the n-π* transition, which happens when electrons move from non-bonding to π* orbitals. There is an increase in UV absorbance when CuO NPs are incorporated.

### Electrical conductivity

**Fig. 7:**
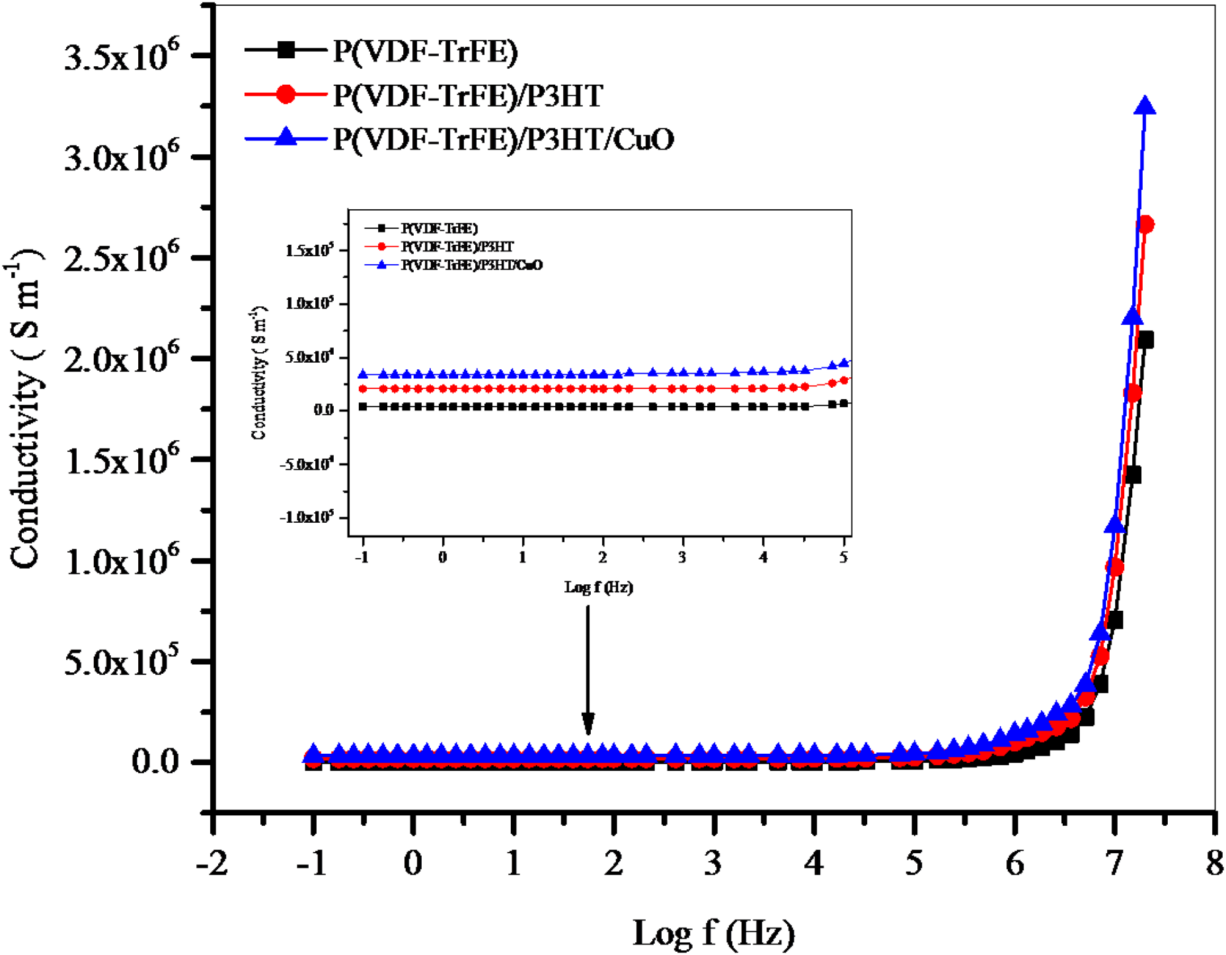
Real part ($\:{\sigma\:}_{ac}$) of AC conductivity of P(VDF-TrFE), P(VDF-TrFE)/P3HT, and P(VDF-TrFE)/P3HT/CuO nanofiber films at 300 K.

Figure 7 shows the frequency dependence of the AC conductivity for P(VDF-TrFE), P(VDF-TrFE)/P3HT, and P(VDF-TrFE)/P3HT/CuO nanofiber films at 300 K. For all nanofiber films, it is observed that at lower frequencies, conductivity stabilizes at constant values, meaning that an increase in frequency does not significantly affect conductivity at low frequencies, reaching its direct current (DC) values. The electrical conductivities of P(VDF-TrFE), P(VDF-TrFE)/P3HT, and P(VDF-TrFE)/P3HT/CuO were obtained 3.75 × 10^3^ S m^− 1^, 2.06 × 10^4^ S m^− 1^, and 3.40 × 10^4^ S m^− 1^, respectively. However, beyond 1 MHz, the conductivity exhibits an exponential relationship with frequency. This behavior is frequently observed in disordered solids, seems to follow the principles of the AC universal law, and is regarded as a strong indication of charge migration through the hopping mechanism. Furthermore, the conduction process in these nanofiber films involves thermally activated localized state hopping. Long-range or slow hopping processes dominate at low frequencies, corresponding to $\:{{\upsigma\:}}_{\text{D}\text{C}}$. Higher frequencies enable shorter hopping distances or higher-energy states, increasing conductivity power-law-like.

### Dielectric properties

**Fig. 8:**
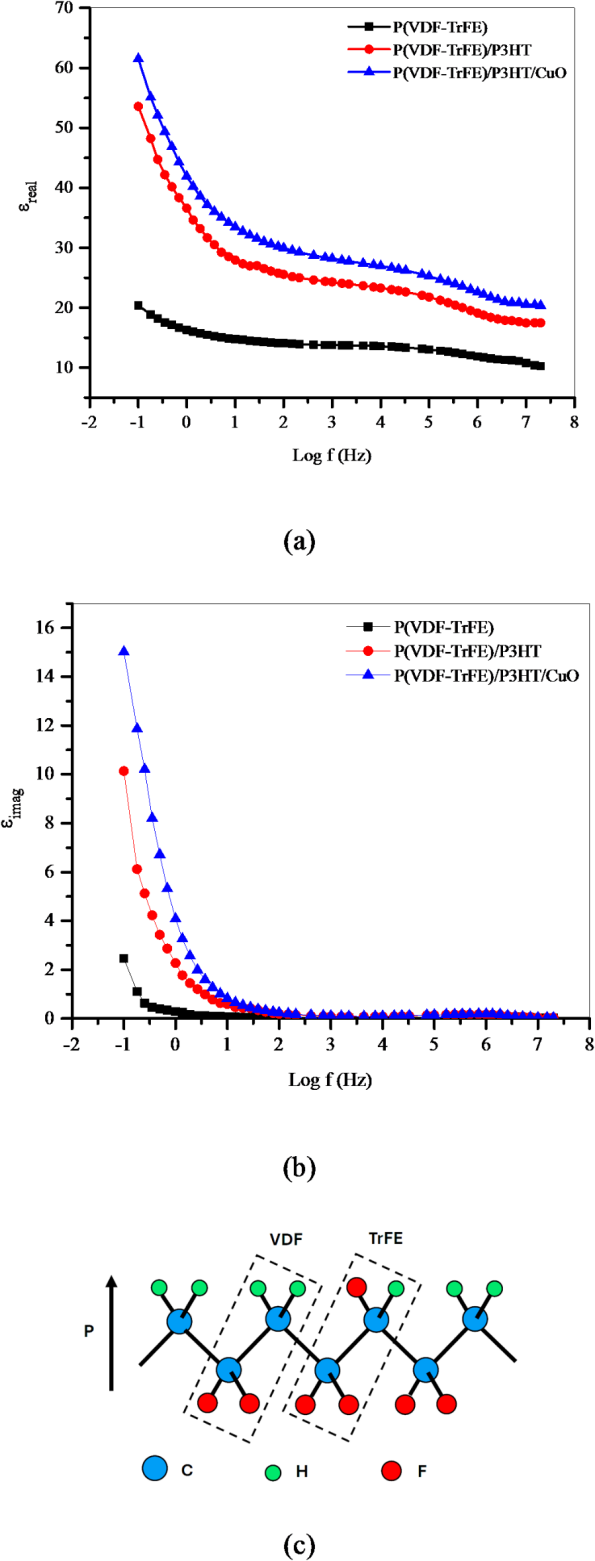
(**a**) Real part, (**b**) imaginary part of the dielectric constant of nanofiber films, and (**c**) schematic representation of β-phase of P(VDF-TrFE).

Figure 8a and b illustrate the relationship between the real and imaginary parts of the dielectric constant and the logarithm of the frequency. It demonstrates that the electrospun nanofiber films exhibit a consistent pattern of decreasing dielectric constant as the frequency increases. A plateau in the dielectric constant was noticed between 10^2^ Hz and 10^4^ Hz. However, at a frequency of 10^2^ Hz, the dielectric constant exhibited a rather high value. This may be attributed to the polarization orientation of the P(VDF-TrFE) chain structure, which is induced by the presence of a permanent dipole moment. Moreover, the dielectric constant of P(VDF-TrFE)/P3HT exhibited a greater value compared to P(VDF-TrFE). Incorporating the semiconducting P3HT into the P(VDF-TrFE) likely caused the improved dielectric characteristics via interfacial polarization at the semiconductor-insulator interface^30^. Two forms of dielectric response exist β-phase rotation and α to β phase transition. In the β-phase, the nearby –CH_2_–CF_2_– dipole aligns parallel to the same direction (Fig. 8c), but in the α-phase, it aligns oppositely^31^. On the other hand, the incorporation of CuO into P(VDF-TrFE)/P3HT increased the value of the dielectric constant. This is because an increase in polarized charges resulting from the dipolar contribution of CuO nanofiller induces polarizability in P(VDF-TrFE)/P3HT when an electric field is applied. The presence of a step-like transition from high to low values of ε_real_ and the observed peak behave as a definitive indication of dielectric relaxations occurring in the nanofiber films. Dielectric relaxation in the nanofiber system is attributed to interfacial polarization and the mobility of the polar segments of polymer chains. The blend of P3HT/CuO induces relaxation in the high-frequency domain.

### Impedance

**Fig. 9:**
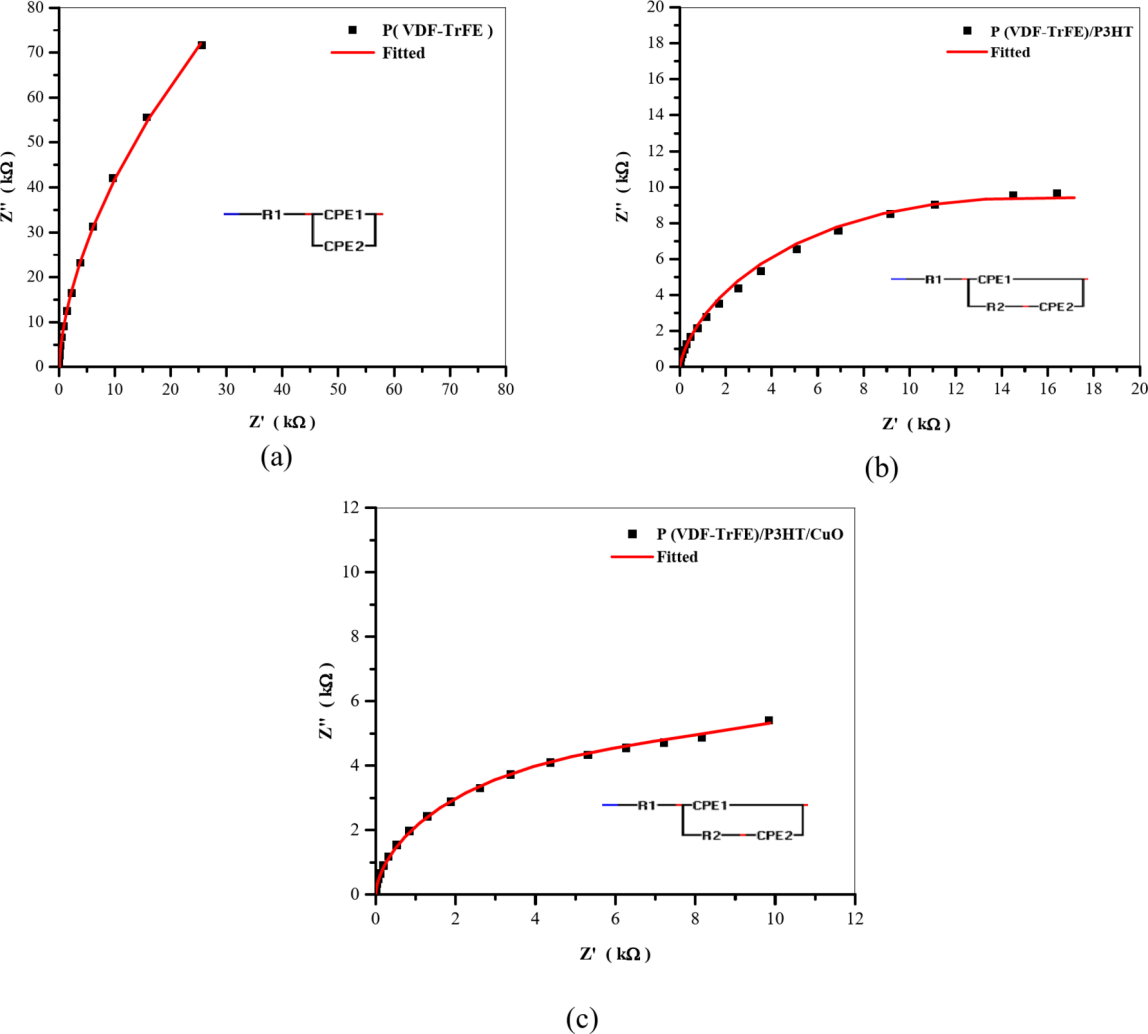
Nyquist plot at room temperature for (**a**) P(VDFTr-FE), (**b**) P(VDFTr-FE)/P3HT, and (**c**) P(VDFTr-FE)/P3HT/CuO electrospun nanofibers.

Figure 9a–c show the impedance plot of electrospun P(VDF-TrFE), P(VDF-TrFE)/P3HT, and P(VDF-TrFE)/P3HT/CuO films at a temperature of 300 K along with their equivalent circuits (shown in inset Fig. 9a–c). The figure is characterized by an inclined straight line over the whole frequency range, which is indicative of the behavior of the electrode/electrolyte double-layer capacitance^32^. The circuits are composed of resistors and constant phase elements (CPE). The CPE’s impedance can be estimated from^33,34^:

Z_CPE_$$\:=\:\frac{1}{\text{Q}\:(\text{i}{\upomega\:}{)}^{\text{n}}}$$ (1)

The element phase, denoted as n, affects the degree of purity deviation exhibited by the capacitor. Q represents the value of 1/|Z|. When *n* = 1, the CPE operates as a pure capacitor; and it functions as a pure resistor at *n* = 0. Based on the curves in Fig. 9a–c; Table 1 summarizes the characteristics of the equivalent circuits model. The bulk resistance can be estimated by the intercepts on the real axis, which have values of 224.5 Ω, 38.3 Ω, and 23.8 Ω for P(VDF-TrFE), P(VDF-TrFE)/P3HT, and P(VDF-TrFE)/P3HT/CuO, respectively. The reduction in bulk resistance suggests the formation of more conductive pathways^35^.

**Table 1:** Fitting parameters of the equivalent circuit.

Nanofiber films	Fitting parameters					
	R1 (Ω)	R2 (Ω)	Q 1 (F)	n1	Q2 (F)	n2
P(VDF-TrFE)	0.224 × 10^3^	–	9.06 × 10^− 12^	0.99	4.57 × 10^− 12^	0.29
P(VDF-TrFE)/P3HT	0.383 × 10^2^	6.74 × 10^2^	4.73 × 10^− 11^	0.23	1.87 × 10^− 11^	0.97
P(VDF-TrFE)/P3HT/CuO	0.238 × 10^2^	2.04 × 10^2^	9.35 × 10^− 11^	0.15	2.14 × 10^− 11^	0.90

## Conclusion

Semiconductive nanofiber films with 10 μm thickness were created using electrospinning techniques. SEM images showed smooth, bead-free solid nanofibers with diameters ranging from 34 to 100 nm. CuO NPs were found to be almost sphere-like and randomly dispersed. XRD pattern reveals the presence of the ferroelectric β phase of P(VDF-TrFE), with a broad diffraction pattern. The FTIR spectrum of the pristine P(VDF-TrFE) fiber film showed distinct peaks for its polar β phase, aromatic C–H groups, and alkyl groups. C=C symmetric stretching and C–C intra-ring stretching dominated the P(VDF-TrFE)/P3HT nanofiber film, compatible with π-electron delocalization. A new diffraction peak at 603 cm^− 1^ may be linked to Cu–O bond vibrations. Incorporating CuO NPs increases UV-Vis absorbance. Conductivity stabilizes at lower frequencies, with an exponential relationship with frequency beyond 1 MHz, indicating charge migration through the hopping mechanism. P(VDF-TrFE)’s electrical conductivity increased from 3.75 × 10^3^ to 3.40 × 10^4^ S m^− 1^ upon the addition of P3HT/CuO. The dielectric constant of P(VDF-TrFE)/P3HT/CuO is higher due to the incorporation of semiconducting P3HT/CuO, which improves dielectric characteristics through interfacial polarization at the semiconductor-insulator interface. The bulk resistance values indicate a reduction in resistance, suggesting more conductive pathways.

## Data Availability

The corresponding author is responsible for providing reasonable access to the datasets used in this study.
